# Complete genomes of *Phascolarctobacterium faecium* isolates obtained from pediatric mucosal-luminal interface aspirate samples

**DOI:** 10.1128/mra.00789-25

**Published:** 2026-01-16

**Authors:** Gillian Tanabe, David R. Mack, Alain Stintzi

**Affiliations:** 1 School of Pharmaceutical Sciences, University of Ottawa 6363, Ottawa, Canada; 2 Department of Biochemistry, Microbiology and Immunology, Ottawa Institute of Systems Biology, Faculty of Medicine, University of Ottawa 6363, Ottawa, Canada; 3 Department of Pediatrics, Faculty of Medicine, University of Ottawa 6363, Ottawa, Canada; 4 Inflammatory Bowel Disease Centre, Children’s Hospital of Eastern Ontario Canada, CHEO Research Institute 274065 , Ottawa, Canada; Nanchang University, Nanchang, Jiangxi, China

**Keywords:** *Phascolarctobacterium faecium*, Crohn's disease, mucosal-luminal interface, gut microbiome

## Abstract

The bacterium *Phascolarctobacterium faecium* frequently colonizes the human gut and has been reported to have reduced abundance in people with Crohn’s disease. Here, we report the complete genome sequences of two *P. faecium* strains isolated from mucosal-luminal interface samples taken from pediatric participants with/without Crohn’s disease.

## ANNOUNCEMENT

*Phascolarctobacterium faecium* is an anaerobic, gram-negative bacterium that frequently colonizes the human gut and is a key succinate consumer and propionate producer (1). *P. faecium* has been found in multiple studies to have reduced abundance in people with Crohn’s disease (2–4). To isolate *P. faecium*, mucosal-luminal interface aspirates (5) from two pediatric participants were anaerobically cultured on 104c PY + X agar supplemented with 80 mM disodium succinate (taken from MediaDive [6]) at 37°C for 4 days. Participants were enrolled within a Children’s Hospital of Eastern Ontario Review Ethics Board approved study (#09/37X). Single colonies were isolated and re-streaked for purity, resulting in strains AB13 from a participant diagnosed with Crohn’s disease and BR41A from a participant not diagnosed with inflammatory bowel disease. Both strains were grown in 104c PY + X broth with 80 mM disodium succinate anaerobically at 37°C for 4 days, followed by centrifugation to pellet the cells. DNA was extracted from the pellets using Quick-DNA HMW MagBead Kits, following the manufacturer’s instructions for microbial lysis, with an additional RNase A treatment and two additional g-DNA Wash Buffer washes (Zymo Research Corp.).

Samples were prepared for Oxford Nanopore sequencing using Rapid Sequencing DNA V14 kits (SQK-RBK114.24) and 200 ng of DNA from each sample. Libraries were size-selected using AMPure XP beads (Beckman Coulter) using a 1:1 bead:DNA ratio and sequenced on R10.4.1 flow cells (FLO-MIN114) on a MinION Mk1C according to the manufacturer’s instructions (Oxford Nanopore Technologies). An Ion Xpress Plus Fragment Library Kit was used to prepare 1 μg of DNA from each sample for short-read sequencing using enzymatic fragmentation as per the manufacturer (Life Technologies). The constructed libraries were size-selected using AMPure XP beads (Beckman Coulter) using a 0.7× bead:DNA ratio, followed by a 0.15× bead:DNA ratio. The libraries were templated on an IonChef with 400 base-read templating and sequenced on an Ion GeneStudio S5 System using an Ion 530 chip with the addition of an Ion S5 calibration standard.

Bioinformatic analyses used the following software with the default parameters, except where specified. Long reads were basecalled and demultiplexed and adapters trimmed using Dorado v0.8.3, e8.2_400bps_hac@v5.0.0 model (github.com/nanoporetech/dorado) followed by filtering using Filtlong v0.2.1 (github.com/rrwick/Filtlong) to discard reads <10,00 bp and the worst 10% of reads. Four programs were used to assemble the long reads: Flye v2.9.5 (7), Canu v2.2 (8), NECAT v0.01-update20200803 (9), and Miniasm v0.3 (github.com/lh3/miniasm). Autocycler v0.2.1 (10) was used to create a consensus assembly from the different assembler outputs. The consensus assemblies for both strains were re-oriented with Dnaapler v1.2.0 (11) to start at the *dnaA* gene and polished with Medaka v2.0.1 (github.com/nanoporetech/medaka). Consensus assemblies were further polished using the short reads with Polypolish v0.6.0 (12) followed by Pypolca v.0.3.1 (12, 13). The genomes were annotated using PGAP v2024-07-18.build7555 (github.com/ncbi/pgap), and completeness of the final assemblies was checked using BUSCO v5.7.0 (14). FastANI v1.1.0 on the Proksee web browser (15, 16) was used to confirm the identity of both strains. Strains AB13 and BR41A both had single circular contigs of lengths 2,260,683 and 2,488,869 bp, respectively (Table 1).

**TABLE 1:** Isolate information, sequencing summary, genome assembly statistics, and accession numbers for both *P. faecium* strains (BioProject PRJNA1275019)

Isolate	AB13		BR41A	
Organism (% average nucleotide identity to NCBI TaxId 1122957)	*P. faecium* (98.96)		*P. faecium* (97.62)	
Sample isolation location	Ottawa, Canada		Ottawa, Canada	
Sequencing technology	Ion S5	ONT	Ion S5	ONT
Total number of raw reads	8,022,157	392,432	23,016,940	414,055
Average raw read length (N50) (bp)	237	4,902 (10,504)	213	5,257 (12,832)
Raw coverage	841×	850×	1,970×	875×
Total number of filtered reads		195,345		184,037
Average filtered read length (N50) (bp)		8,863 (11,807)		10,644 (14,555)
SRA accession number	SRR33969888	SRR33969887	SRR33969891	SRR33969890
		SRR33969886		SRR33969889
BioSample	SAMN49008379		SAMN49008380	
GenBank accession number	CP195761		CP195918	
BUSCO completeness (%)	97.7		97.3	
Genome size (bp)	2,360,683		2,488,869	
GC content (%)	43.9		43.5	
Genes	2,206		2,443	
Coding sequences (CDSs)	2,131		2,368	
Transfer RNAs (tRNAs)	56		56	
Non-coding rRNAs (ncRNAs)	4		4	
Ribosomal RNAs (rRNAs)	15		15	
Pseudogenes	8		11	

## Data Availability

The BioProject for AB13 and BR41A is PRJNA1275019. Raw sequencing reads were deposited in the Sequence Read Archive for AB13 under accessions SRR33969888, SRR33969887, SRR33969886; and for BR41A under accessions SRR33969891, SRR33969890, SRR33969889. The genome assemblies for AB13 and BR41A have been deposited in GenBank under accession numbers CP195761 and CP195918.
